# The Effects of Exergaming on Attention in Children With Attention Deficit/Hyperactivity Disorder: Randomized Controlled Trial

**DOI:** 10.2196/40438

**Published:** 2023-05-09

**Authors:** HongQing Ji, Shanshan Wu, Junyeon Won, Shiyang Weng, Sujin Lee, Sangmin Seo, Jung-Jun Park

**Affiliations:** 1 School of Physical Education & Health Wenzhou University Wenzhou China; 2 Division of Sport Science Pusan National University Busan Republic of Korea; 3 Institute for Exercise and Environmental Medicine Texas Health Presbyterian Hospital Dallas, TX United States; 4 Busan Children's Mind Clinic Busan Republic of Korea

**Keywords:** exergame, N2 amplitude, attention function, response time, attention deficit/hyperactivity disorder, ADHD

## Abstract

**Background:**

Despite growing evidence showing the effects of exercise and cognitive trainings on enhancing attention, little is known about the combined effects of exergame on attention in children with attention deficit/hyperactivity disorder (ADHD). Exergame, a form of exercise using a video game, has both cognitive stimulation and physical activity components and has been shown to improve cognitive function in children.

**Objective:**

The purpose of this study was to investigate the effect of exergaming on attention and to compare the effect induced by exergaming with the effect of aerobic exercise on attention in children with ADHD.

**Methods:**

In all, 30 children with ADHD, aged 8-12 years, were randomly divided into an exergaming group (EXG; n=16) or a bicycle exercise group (BEG; n=14). Before and after the 4-week intervention, the *Frankfurter Aufmerksamkeits-Inventar* (FAIR; Frankfurt Attention Inventory) test was administrated, and event-related potentials during the Go/No-go task was measured to assess attention.

**Results:**

After intervention, both the EXG and BEG had significantly increased selective attention and continuous attention (all *P*<.001), as well as self-control on the FAIR test (EXG: *P*=.02 and BEG: *P*=.005). Similarly, both the EXG and BEG had significantly reduced response time on the Go/No-go test (all *P*<.001). For the Go response, the N2 amplitude (frontocentral maximal negativity) was significantly increased in Fz (midfrontal line) in the EXG (*P*=.003) but was not changed in the BEG (*P*=.97). Importantly, the N2 amplitude in Fz was significantly greater in the EXG compared to the BEG (Go: *P*=.001 and No-go: *P*=.008).

**Conclusions:**

Exergaming has the comparable effects to bicycle exercise to enhance attention in children with ADHD, suggesting that exergaming can be used as an alternative treatment for children with ADHD.

**Trial Registration:**

Clinical Research Information Service KCT0008239; https://tinyurl.com/57e4jtnb

## Introduction

Attention deficit/hyperactivity disorder (ADHD) is a neurodevelopmental disorder that is characterized by inattention, hyperactivity and impulsivity, or both [1]. Approximately 3% to 5% of the school-age population are living with ADHD [2,3]. Among symptoms of ADHD, hyperactivity tends to improve during adolescence, but carelessness and impulsiveness may persist into adulthood [4]. The impaired attention and executive function in children with ADHD lead to reduced inhibitory ability, working memory, and task shift due to an imbalance of neurotransmitters such as dopamine and norepinephrine [5-7]. The abnormal neuropsychological activities caused by these symptoms may disrupt the patients’ learning ability, daily activities, professional activities, and social functions [8,9].

In children with ADHD (aged >8 years), dysfunctions in neuronal networks associated with attentional processes and cognitive control have been well documented using event-related potentials (ERPs) [10-13]. ERP studies used tasks (eg, Eriksen flanker and Go/No-go tasks) to assess both behavioral and neurophysiological perspectives of ADHD [14]. For example, behavioral performance (eg, response time [RT]) is commonly used to determine processing speed [15], and neurophysiological technology has been used to measure selective attention capacity and skills [16]. The No-go N2 components have been reported to be abnormally lower in children with ADHD than those in children with other neurodevelopmental disorders [17,18]. The N2 amplitude occurs for about 170-350 milliseconds and is related to attention and cognitive control, specifically an inhibitory control response [16,19,20]. Particularly, the amplitude of the N2 is an indication of the intensity of information processing required for the discrimination of stimuli, and the latent phase of the N2 reflects the time of the cognitive processing that distinguishes sensory stimuli [21].

Exercise has been known to enhance attention, executive function, impulsivity, and hyperactivity in children with ADHD by promoting the secretion of neurotransmitters (eg, dopamine and norepinephrine) in the brain, thereby inducing positive responses on the main symptoms of ADHD [22,23]. Although most of the evidence has focused on the acute effects of aerobic exercise on RT and P3 components of children with ADHD, N2 components have received comparatively little attention. Furthermore, there are mixed results in the study of exercise and N2 in normal young adults and preadolescent children [24-26]. For example, Ligeza et al [24] reported that acute moderate intensity exercise increased the conflict effect of N2 amplitude in young people, which suggested improvement in inhibition after a single session of moderate exercise compared to high-intensity interval exercise and seated rest condition. In another study, acute moderate-intensity exercise led to reduced N2 amplitude in preadolescent children and young adults, suggesting that acute exercise might have a general effect on the conflict detection process [25,26]. Therefore, further studies are needed to explore whether exercise can improve the N2 components of children with ADHD.

However, previous studies reported that cognitively engaging physical exercise leads to benefits for cognitive performance [27,28]. For example, a combination of physical activity and cognitive stimulation improved reaction times in inhibition and switching neural efficiency [27,28]. Exergaming is a video game that requires body movement and provides active gaming experience as a form of physical activity [29]. Due to motivational issues and a low level of positive reinforcement, traditional (cognitive) training programs are often uninteresting and fatiguing for children with ADHD [30]. Exergaming helps combine physical and cognitive training through an intriguing game. Recently, exergaming has attracted great attention because of their growing popularity among children, adolescents, and older adults [31-35] due to its effectiveness in motivating participants to engage in intervention [36].

Recent studies revealed that exergaming improves the executive function in healthy adolescents by increasing cognitive stimulation [31]. Exergaming has also been reported to enhance executive function in children with ADHD compared to the control group [37,38]. However, it is currently in question whether these effects are caused by games or exercise. Moreover, there is, to date, only one study that has measured the effects of exergaming on N2 amplitude. This study showed that exergaming led to greater aerobic capacity and larger N2 amplitude, an indication of improved selective attention in patients with metabolic syndrome [39]. Despite the evidence regarding the effects of exergaming on cognitive function, how the neural processing of N2 components is altered in children with ADHD after an exergaming intervention remains unclear. Moreover, it remains inconclusive if exergaming improves attention in children with ADHD and whether exergaming has additional benefits compared to traditional form of aerobic exercise. Therefore, the purpose of this study was to investigate the effect of exergaming on attention and to compare the effect induced by exergaming with the effect of aerobic exercise on attention in children with ADHD. Our first hypothesis was that exergaming will result in improved Go/No-go task performance and increased N2 amplitude in children with ADHD. Second, we hypothesized that the improvement in Go/No-go task performance and N2 amplitude will be greater in exergaming compared to bicycle exercise.

## Methods

### Study Design and Participants

We used a randomized controlled trial in which participants were randomly assigned to either the exergaming group (EXG) or the bicycle exercise group (BEG). Children with mild to moderate ADHD were recruited from the Children’s Mental Health Medical Center in Busan, Republic of Korea. Participants who met the following criteria were included in the study: a Korean Attention-Deficit/Hyperactivity Disorder Diagnostic Scale score between 70-110, indicating a lack of attention; the absence of diseases other than ADHD; right-hand dominance; normal or corrected-to-normal vision; and the absence of physical impairment to perform exercise.

**Figure 1:**
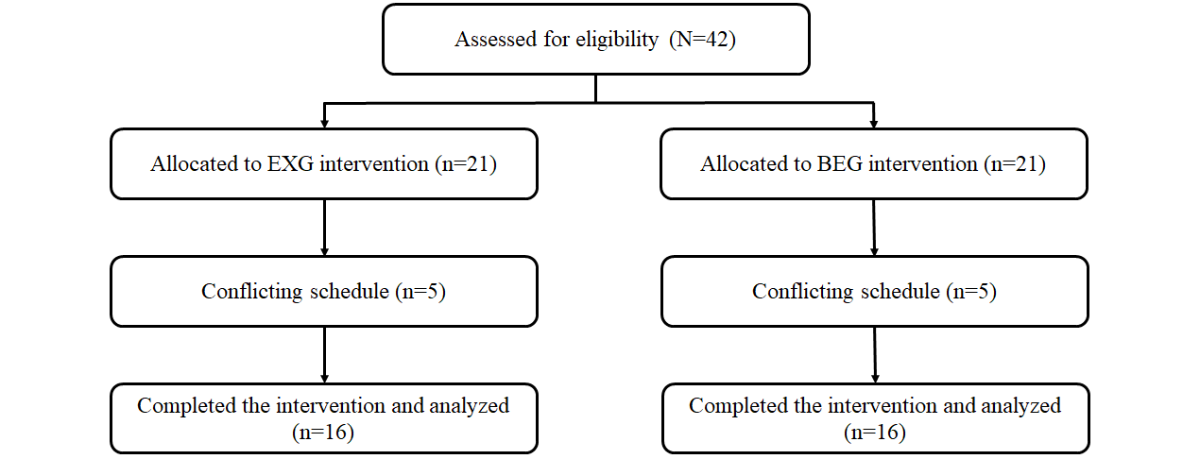
Flowchart of participant eligibility, withdrawals, and the final sample included in the final analysis. BEG: bicycle exercise group; EXG: exergaming group.

In all, 42 children (aged 8-12 years) with mild to moderate ADHD were recruited and then randomly assigned to either the EXG (n=21) or BEG (n=21). A total of 12 participants (5 for the EXG and 7 for the BEG) were withdrawn from the study, resulting in the final number of 30 participants in this study (16 in the EXG and 14 in the BEG; Figure 1). All participants maintained the same medication and dose during the exercise intervention.

### Ethics Approval

Written informed consent was obtained from both the children and their parents or legal guardians. This study was approved by the Pusan National University Institutional Review Board in accordance with the Helsinki Declaration (PNU IRB/2018_30_HR).

### Sample Size

The sample size was calculated using a sample size calculation software program (G*Power, version 3.1.9.2 for Windows), with an effect size of 0.58, statistical power of .80, and statistical level of significance of .05. The effect size was calculated from previous studies [40]. As a result, the sample size for each group was established at 15 patients, so we decided to recruit 42 patients for each group in consideration of a potential 30% dropout rate.

### Exergaming and Bicycle Exercise Intervention

**Figure 2:**
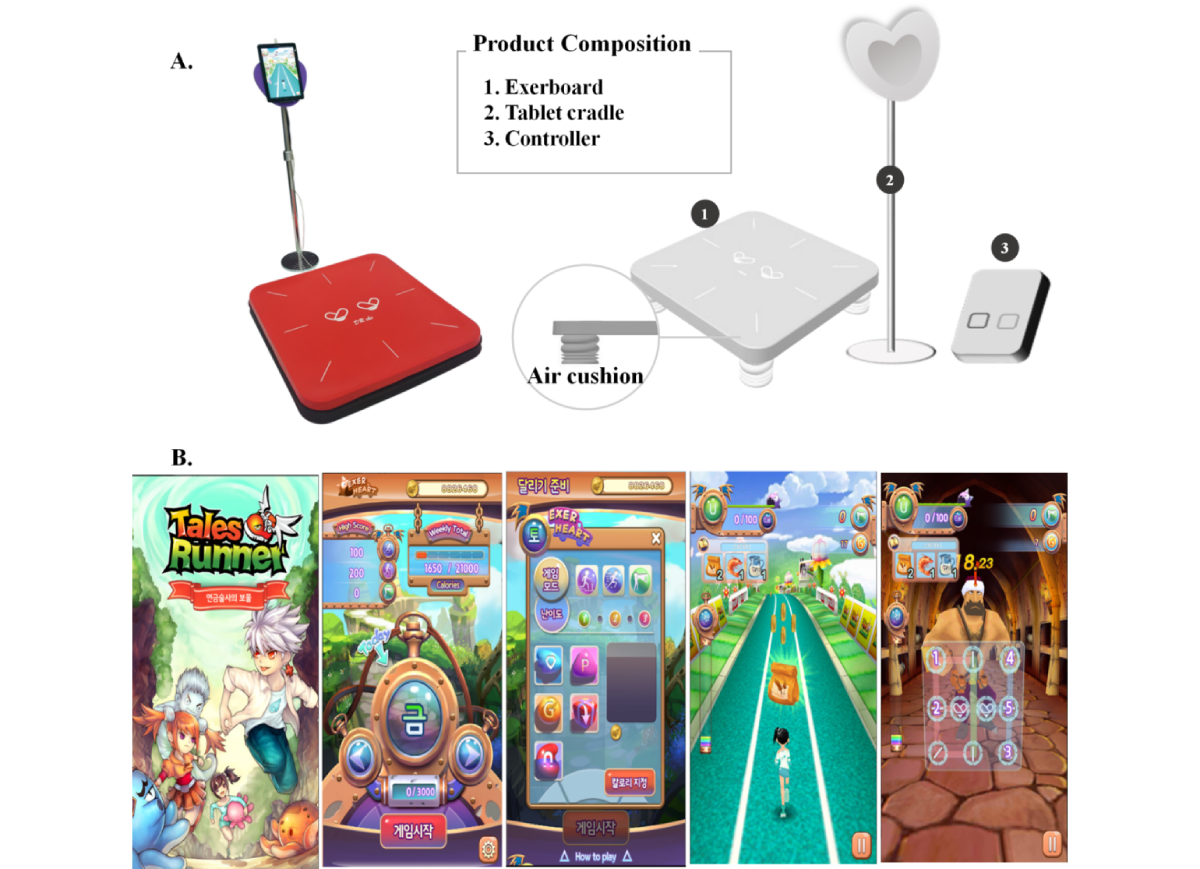
(A) ExerHeart devices consisted of the Exerboard, a tablet cradle, and a controller. (B) Features of the video game Alchemist's Treasure.

The EXG was administered using ExerHeart devices (D&J Humancare), which consisted of a running or jumping board (730-cm width × 730-cm depth × 130-cm height) and a screen connected to the board (Figure 2A). The exercise program called *Alchemist’s Treasure* (D&J Humancare) was used for the EXG. In this game, participants run or jump in place with their avatars, using the front, back, left, and right sensors on the mat to avoid obstacles and acquire items (Figure 2B; Multimedia Appendix 1). The BEG performed stationary bike exercise using commercial Fit Elite-Whole body exerciser 1000, with resistance of 0.5~3 kiloponds.

Exercise session for both the EXG and BEG consisted of 3 days/week, 50 min/day, and 60% to 80% of heart rate (HR) reserve for 4 weeks. We monitored individual exercise intensity using an HR monitor (Polar RS400sd). Exercise intensity was determined by using the Karvonen target HR method: *[exercise intensity × (HR_max_ – resting HR)] + resting HR* [41]. Each exercise session consisted of 10 minutes of warm-up, 30 minutes of main exercise, and 10 minutes of cooldown. Exercise interventions were conducted at 2 separate child mental health care facilities so that the groups were not aware of the exercise program they were performing. Participants who did not complete more than 80% of the exercise sessions were excluded from the final analysis.

For both the EXG and BEG, all subjects’ HRs during exercise were monitored using HR monitors (Polar RS400sd) to confirm that the values were within the target HR range.

### Go/No-go Task

The Go/No-go task was used to measure the capacity to sustain attention and response control. We used the computerized Go/No-go task using a built-in software of the electroencephalography (EEG) analyzer (Telescan; LAXTHA). During the Go/No-go task, each participant was instructed to press a button for a frequent stimulus (ie, Go stimulus, 60% probability) or to withhold a response for an infrequent stimulus (ie, No-go stimulus, 40% probability). The stimulus was presented in the center of a 15-inch screen with a white background, and the size was 3 × 3 cm. In each trial, a signal consisting of an interstimulus interval was first presented for 1000 milliseconds to attract the participant’s attention. This signal was followed by either the Go stimulus (ie, lion image), No-go stimulus (ie, tiger image), or Control stimulus (ie, leopard image) for 1500 milliseconds. Participants were instructed to press the left arrow keyboard for the Go stimulus and the right arrow keyboard for the No-go stimulus and not to respond to the Control stimulus. Participants underwent a familiarization session before starting the test. The number of practice trials was standardized (1 trial each) so that participants performed the same number of practice trials before each condition. The response time (milliseconds) and the accuracy rate (% correct) were recorded simultaneously from the start of the reaction. The entire task (including practice trials) lasted for 10 minutes.

### EEG Measurements

EEG was measured from 31 Ag/Ag–CL electrodes, placed according to an augmented 10–20 International System. All EEG recordings were referenced to the average of the right and left mastoid, and the ground electrode was placed on the Fz electrode site. The electrooculogram activity was recorded from electrodes attached below and above the left eye and electrodes located at the outer canthi of both eyes. All electrodes were maintained at impedances <10 kΩ before data recording. After the completion of data collection, EEG signals were analyzed using software (Telescan; LAXTHA); the bandpass filter of the amplifier was 0.5-20 Hz, the sampling rate was 1000 Hz, and a notch filter was at 60 Hz.

The stimulus-locked epochs acquired for the Go/No-go test were extracted offline from 200 milliseconds before to 1500 milliseconds after the stimulus onset, and the period from –100 to 0 milliseconds before stimuli onset was used as the baseline. Peak amplitudes and latencies were measured automatically. The N2 peak amplitudes and latencies were measured in the difference waves of the attended condition. The N2 component was defined as the largest positive peak occurring between 170-320 milliseconds after stimuli. EEG data were collected before and after 8 weeks of the exercise intervention [42,43].

### Frankfurter Aufmerksamkeits-Inventar

The *Frankfurter Aufmerksamkeits-Inventar* (FAIR; Frankfurt Attention Inventory) is a psychological test to assess attention and concentration [44]. The FAIR tests the ability to quickly distinguish correct signal among many other similar signals and to hide insignificant information (ie, external interference). Two different versions of the FAIR test were used for test reliability. A total of 640 test items were divided into tests 1 and 2. Each FAIR test paper was arranged in the shape of 20 horizontal and 16 vertical lines on a single sheet of paper, and there were a total of 320 test items. Participants were instructed to find 2 correct shapes, such as a “three-point circle” and “two-point square” among the 4 shapes and draw a line from left to right with a pencil on the test paper to mark them. The scoring was administered using the perspective of 3 attentional actions, and details are shown in Table 1. The ability index P is an index of the number of items related to attention during the test. The control index Q represents the proportional value of correct judgment as a result of attention among all responses. The persistence index C indicates how consistently the attention task was maintained.

**Table 1:** *Frankfurter Aufmerksamkeits-Inventar* inspection dimensions.

Test item	Related ability	Scoring method	Reliability
P^a^	Selective attention	*(T^b^ – EL^c^) – 2(EO^d^ + EC^e^)*	.944
Q^f^	Self-control	*P ÷ T*	.903
C^g^	Sustained attention	*P × Q*	.941

^a^P: performance value.

^b^T: total number of items worked.

^c^EL: total number of line drawing errors.

^d^EO: total number of teeth not marked on a target item.

^e^EC: total number of teeth marked that are not a target item.

^f^Q: quality value.

^g^C: continuity value.

### Statistical Analysis

The normality of Go/No-Go test performance was also tested using the Shapiro-Wilk test. Repeated measures ANOVA were used to compute the main effects of time (ie, before vs after intervention), group (ie, EXG vs BEG), and group × time interaction on the behavioral performance (ie, accuracy rate and reaction time), ERP (ie, N2 amplitude), and FAIR (ie, ability index P, control index Q, and persistence index C). If an interaction was identified, paired sample *t* test (2-tailed) was used to verify the direction of the interaction. Significance level was set at .05 for all analyses, effect sizes were assessed using partial eta squared (η^2^_p_), and all statistical analyses were performed using SPSS (version 24; IBM Corp).

## Results

### Participants

Of the 42 children who participated in this study, 5 in the EXG dropped out of the study due to conflicting schedule and 7 in the BEG dropped out of the study due to lost interest. Demographic and physical characteristics for all subjects are provided in Table 2.

**Table 2:** Summary of the participant demographic information.

	EXG^a^ (n=16)	BEG^b^ (n=14)
Age (years), mean (SD)	9.00 (1.46)	8.85 (1.63)
Male sex, n (%)	14 (88)	12 (86)
Height (cm), mean (SD)	129.38 (9.68)	134.69 (7.76)
Weight (kg), mean (SD)	31.66 (11.84)	35.31 (6.84)
BMI (kg/m^2^), mean (SD)	18.5 (5.4)	19.3 (2.7)
K-ADHDDS^c^ (index), mean (SD)	96.14 (19.16)	87.50 (13.17)

^a^EXG: exergaming group.

^b^BEG: bicycle exercise group.

^c^K-ADHDDS: Korean Attention-Deficit/Hyperactivity Disorder Diagnostic Scale.

### Behavioral Indices

**Figure 3:**
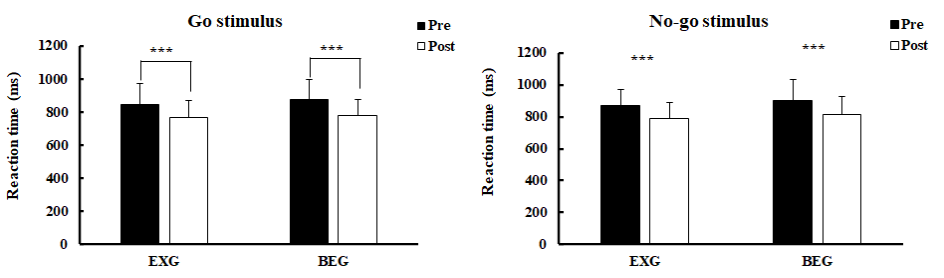
Reaction time during Go/No-go task in the EXG and BEG before and after exercise intervention (A) Go stimulus, (B) No-go stimulus. BEG: bicycle exercise group; EXG: exergaming group. ***P<.001.

There was no significant group × time interaction on the changes in RT in response to Go and No-go stimulations before and after exercise intervention (*P*=.65 and *P*=.807, respectively). However, both groups significantly reduced RT for Go and No-go stimulations after exercise intervention (Figure 3). For Go stimulation, RT was significantly reduced in the EXG from 846.13 (SD 126.43) milliseconds to 764.56 (SD 107.05) milliseconds (*P*=.001; η^2^_p_=0.969) and in the BEG from 873.79 (SD 123.31) milliseconds to 780.79 (SD 96.41) milliseconds (*P*=.001; η^2^_p_=1.267; Figure 3A). For No-go stimulation, RT was significantly reduced in the EXG from 872.25 (SD 96.53) milliseconds to 786.75 (SD 100.02) milliseconds (*P*=.001; η^2^_p_=1,211) and in the BEG from 903.71 (SD 130.78) milliseconds to 813.77 (SD 111.86) milliseconds (*P*=.001; η^2^_p_=1.114; Figure 3B).

### N2 Amplitude

**Figure 4:**
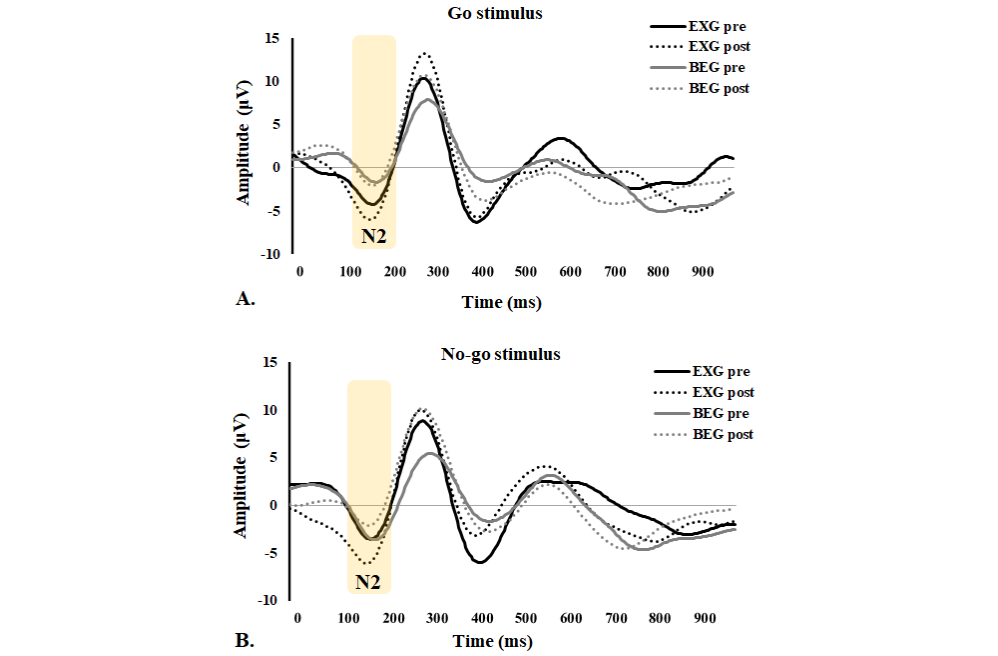
Average event-related potential waveforms of Fz for mean N2 amplitudes during the Go/No-go test: (A) Go stimulus and (B) No-go stimulus in the exergame group (EXG) and bicycle exercise group (BEG) before and after the exercise intervention.

There was a significant group × time interaction on the Go and No-go N2 amplitudes in the Fz region before and after exercise intervention (*P*=.049 and *P*=.047, respectively; Table 3). The EXG had significantly increased Go N2 amplitude in Fz after intervention (*P*=.003), whereas the BEG showed no significant changes (*P*=.97). Neither group showed changes in No-go stimulus after intervention. In the between-group comparison, the EXG consistently demonstrated greater N2 amplitudes on both Go and No-go stimulations. The waveforms of Go and No-go N2 amplitudes in Fz for the EXG and BEG before and after exercise are shown in Figure 4.

**Table 3:** Results of changes in Go/No-go N2 amplitude in Fz.

Effects and group					Preintervention			Postintervention			*t* test					*P* value										
										*P* value			η^2^_p_		G^a^				T^b^			G×T^c^			η^2^_p_	
**Go** **stimulus**																	.02			.054			.049			.08
	EXG^d^, mean (SD)			–3.92 (3.78)			–6.82 (4.49)			.003			1.791													
	BEG^e^, mean (SD)			–2.33 (2.15)			–2.30 (4.05)			.97			0.020													
	***t*** **test**																									
		*P* value	.18			.008																				
		η^2^_p_	0.503			1.045																				
**No-go** **stimulus**																	.008			.68			.047			.08
	EXG, mean (SD)			–4.64 (3.65)			–6.70 (3.85)			.09			0.911													
	BEG, mean (SD)			–3.55 (3.91)			–2.18 (2.36)			.27			0.615													
	***t*** **test**																									
		*P* value	.44			.001																				
		η^2^_p_	0.289			1.345																				

^a^G: group effect.

^b^T: time effect.

^c^G×T: interaction effect between group and time.

^d^EXG: exergaming group.

^e^BEG: bicycle exercise group.

### Aufmerksamkeits-Inventar Data

There was no significant group × time interaction on the ability index P (selective attention) before and after exercise intervention (*P*=.79). However, both groups significantly increased ability index P after exercise intervention (both *P*<.001). Similarly, although there was no significant interaction between the EXG and BEG on the on the changes in ability index Q (self-control; *P*=.68), both the EXG and BEG significantly increased control index Q following intervention (EXG: *P*=.02 and BEG: *P*=.005). Lastly, there was also no significant interaction between the EXG and BEG on the on the changes in ability index C (persistent attention; *P*=.66), but both groups significantly increased index C in response to exercise intervention (both *P*<.001; Table 4).

**Table 4:** Results of changes in *Frankfurter Aufmerksamkeits-Inventar* (FAIR).

Effects and group				Preintervention		Postintervention		*t* test			*P* value							
							*P* value		η^2^_p_	G^a^			T^b^		G×T^c^		η^2^_p_	
**Performance value (P; score)**												.19		.001		.79		.10
	EXG^d^, mean (SD)		190.44 (47.85)		269.06 (92.13)		.001		2.070									
	BEG^e^, mean (SD)		231.15 (71.44)		303.92 (101.78)		.001		2.308									
	***t*** **test**																	
		*P* value	.08		.34													
		η^2^_p_	0.670		0.354													
**Quality value (Q; ratio)**												.19		.001		.68		<.001
	EXG, mean (SD)		0.90 (0.08)		0.95 (0.005)		.02		1.339									
	BEG, mean (SD)		0.92 (0.06)		0.97 (0.02)		.005		1.833									
	***t*** **test**																	
		*P* value	.31		.23													
		η^2^_p_	0.381		0.451													
**Continuity value (C; score)**												.20		.001		.66		.17
	EXG, mean (SD)		171.33 (48.30)		262.44 (98.58)		.001		2.070									
	BEG, mean (SD)		214.66 (76.67)		294.70 (10(3.07)		.001		2.308									
	***t*** **test**																	
		*P* value	.07		.40													
		η^2^_p_	0.679		0.314													

^a^G: group effect.

^b^T: time effect.

^c^G×T: interaction effect between group and time.

^d^EXG: exergaming group.

^e^BEG: bicycle exercise group.

## Discussion

### Principal Findings

The purpose of this study was to examine the effects of exergaming on attention in children with ADHD and whether these effects were driven by games or exercise by comparing the effects of exergaming and bicycle exercise. Through an examination of the behavioral performance and ERP during the Go/No-go task and FAIR test, our results may suggest that both exergaming and bicycle exercise elicited shorter RT during the Go/No-go task and improved selective attention, self-control, and persistent attention. Notably, larger N2 amplitudes were observed following exergaming compared to the bicycle exercise.

Our results are in agreement with prior findings that showed improved processing speed in children with ADHD after exercise. For example, acute aerobic exercise engendered significantly faster processing speed during the Stroop and flanker tasks compared to the control group [45]. In another study, an 8-week yoga program enhanced attention and processing speed in children with ADHD [46]. Research shows that exercise facilitates the upregulation of cerebral blood flow (CBF) [47] and that an exercise-induced increase in CBF promotes information processing speed [48]. Further, a possible neurophysiological mechanism underpinning the beneficial effects of exercise training on attention in children with ADHD was the exercise-induced secretion of catecholamine [49]. Therefore, in this study, it may be that both exergaming and bicycle exercise stimulate CBF increase and catecholamine secretion, thereby promoting the allocation of attention resources and improvement of processing speed in children with ADHD. However, since this study did not measure CBF and catecholamines levels, this interpretation should be viewed with caution, and the potential neurophysiological mechanism underpinning exergaming and attention should be further investigated in the future.

Novel to this investigation is that there was an interaction effect of exercise modes on changes in the N2 amplitude during the Go/No-go task after intervention, such that the improvements in the N2 amplitude were only manifested in exergaming while the intervention effects were not observed in the bicycle exercise. The N2 components provide important information through the amplitude and latent phase because it is an indication of attentional assignment of the anterior cingulate cortex (ACC) when performing cognitive tasks [50,51]. Baker and Holroyd [52] suggested that task demands involving high conflict and working memory loads should strongly activate the ACC, giving rise to increased N2 amplitudes. No-go stimulation activates the prefrontal cortex and can affect cognitive regulation and inhibition processes [53]. Previous studies have reported that the N2 amplitude reflects the neural changes associated with the task [54] and that the N2 amplitude increases with greater attention [51]. Drollette et al [25] reported that the N2 amplitude decreased after running on the treadmill for 20 minutes, but another study showed that the N2 amplitude remained unchanged after an acute exercise [55]. Additionally, Stroth et al [56] pointed out that greater physical fitness is associated with better task preparation as well as decreased amplitudes in N2 amplitude, indexing more efficient executive control processes. In our previous study, both exergaming and treadmill exercise improved selective attention (N2) in patients with metabolic syndrome [39]. The results indicated that exergaming facilitated visual perceptual stimulation in the virtual environment to enhance the selective attention activity within with the cerebral cortex, brain regions associated with executive function, which was not changed by normal aerobic exercise.

In light of our N2 amplitude findings, exergaming is likely to better regulate the activity of ACC in the ventral prefrontal cortex than traditional form of aerobic exercise and effectively increase attention. It is hypothesized that ACC activation may have been reduced due to less-engaging exercise environment, which engendered no beneficial effect on attention after bicycle exercise. This may also be associated with the fact that all participants who dropped out from the BEG (n=7) were due to lost interest, whereas all participants who dropped out from the EXG (n=5) were due to conflicting schedule. This obvious discrepancy between the groups in the dropout reasons occurred even though the interventions were administered in 2 separate child mental health care facilities, which was intended to avoid environmental factors that could influence the participants’ motivation. Thus, the ACC is likely to be more actively stimulated during complex exercise that involves multiple aspects of environment (eg, exergaming) than monotonous exercise environment (eg, cycling). Performing exergaming requires a process of directional judgment (eg, front or back and left or right); thus, attention and cognitive control ability needs to be executed to successfully perform the task. However, further translational evidence is needed to clarify this hypothesis.

Interestingly, aerobic exercise has a faster judgment of response times when it comes to information processing, but cash realization during neuroelectrical activity has a different effect. It may be that in the applied exergaming, the inhibition and switching components are needed to a greater extent than updating for successful task performance [37]. The Alchemist’s Treasure training program primarily includes strength training; coordination (and endurance); sensitivity training; as well as demands on cognitive functions such as inhibition, switching, updating, attention, and rapid action execution. Prior research has demonstrated that cognitively demanding physical activities (exergaming), such as coordination exercises, preferentially activate brain regions used to control higher-order cognitive processes, leading to improved performance [31,37]. In conclusion, exergaming has the characteristics of aerobic exercise, so it has the ability to judge reaction time quickly. Meanwhile, the brain area is stimulated by the corresponding stimulation more favorable to neuroelectrical activity than general aerobic exercise.

For the FAIR results, we found improved selective attention, self-control, and persistent attention following exergaming and bicycle exercise. The results of Medina et al [57] showed that physical activity increases the release of serotonin, dopamine, and norepinephrine, and it is assumed that the attention of the participants with ADHD is improved because of the release of these neurotransmitters. According to a previous investigation, tai chi training was conducted to investigate the attentional in children and adolescents with ADHD [58]. The results showed that continuous tai chi performance at a stable speed significantly enhanced the ability of selective attention and continuous attention. Studies in adolescents with ADHD have shown that exercise-based game intervention confers maximal benefits for selective attention, self-control, and persistent attention [59,60].

We surmise that 3 components of executive function (ie, selective attention, self-control, and persistent attention) have been facilitated during exergaming performance. First, selective attention is the process of stimulating specific consciousness. During exergaming, participants had to quickly avoid the obstacles that appear on the screen; thus, participants had to maintain their selective attention throughout the game. The second component is cognitive control, which is necessary to complete tasks within a certain period of time and is expected to be facilitated while pressing the displayed number on the screen quickly and accurately during exergaming. Lastly, sustained attention is the duration of concentration related to the duration of correct concentration. In this study, exergaming characters were controlled by the user’s movement. To control the game character’s movement accurately and quickly, participants had to maintain their attention throughout the game. Therefore, because exergaming contains general aerobic exercise elements as well as additional components to stimulate selective attention, self-control, and continuous attention, it may have had significant impacts on the attention and attention of children with ADHD.

### Limitations

Our study is not without limitations. First, this study is subject to the limitation of lacking a nonexercise (or active) control group, warranting some caution in interpreting the results until they can be replicated in a larger randomized controlled trial. Second, this study is also limited by a relatively small sample size (n=30) and homogeneous characteristics of participants (ie, all Asian and 26/30, 87% male), so the results may not be generalizable to the entire population of children with ADHD. Third, only one type of game device was used in this study; thus, future studies also need to examine the effects of multiple exergaming devices and games on ADHD attention. Fourth, the relationship between game scores and attention was not considered in this study, which should be examined in future studies.

### Conclusions

Our results showed that both exergaming and bicycle exercise training enhanced attention in children with ADHD, but the benefits induced by exergaming may be somewhat greater than those from bicycle exercise. These findings suggest that, through complex and stimulating environment during the training, exergaming may stimulate the frontal lobes of the brain and has a significant impact on attention, processing speed, and cognitive control function, thereby improving attention in children with ADHD symptoms. Therefore, this study may hold an important public health implication that exergaming could be used as a new exercise therapy for children with ADHD in the future.

## Appendix

Multimedia Appendix 1 Participants played the video game with ExerHeart.

Multimedia Appendix 2 CONSORT-EHEALTH checklist (V 1.6.1).

## Abbreviations

ACC: anterior cingulate cortex
ADHD: attention deficit/hyperactivity disorder
BEG: bicycle exercise group
CBF: cerebral blood flow
EEG: electroencephalography
ERP: event-related potential
EXG: exergaming group
FAIR: Frankfurter Aufmerksamkeits-Inventar (Frankfurt Attention Inventory)
HR: heart rate
RT: response time
